# Effects of nanoparticles on laser sintering of metal and ceramic powders

**DOI:** 10.1557/s43580-025-01375-9

**Published:** 2025-09-10

**Authors:** Jovana Latinovic, Ka’Tra Winchester, Noble Agyeman-Bobie, Zie-Nia Rice, Stecy Chirinda, Haeyeon Yang

**Affiliations:** Department of Mathematics and Physics, Grambling State University, Grambling, LA 71245 USA

## Abstract

Continuous wave (CW) laser was used to melt micrometer-sized powders mixed with nanoparticles (NPs) such as titanium NPs. Nanoliquids have been known as medium of excellent heat transfer so that commercial nanoliquids are available for more efficient cooling engines and drill bits for oil wells. Nanoliquids may form during the laser melting or sintering of powders that are mixed with NPs of higher melting point than that of powders. During the melting period, NPs may stay in solid phase while the powders become liquid. This powder-transformed to liquid may be viewed as a nanoliquid with solid-state NPs. We discuss the laser melting of powders such as SmCo_5_ with titanium NPs including their hardness.

**Graphical abstract:**

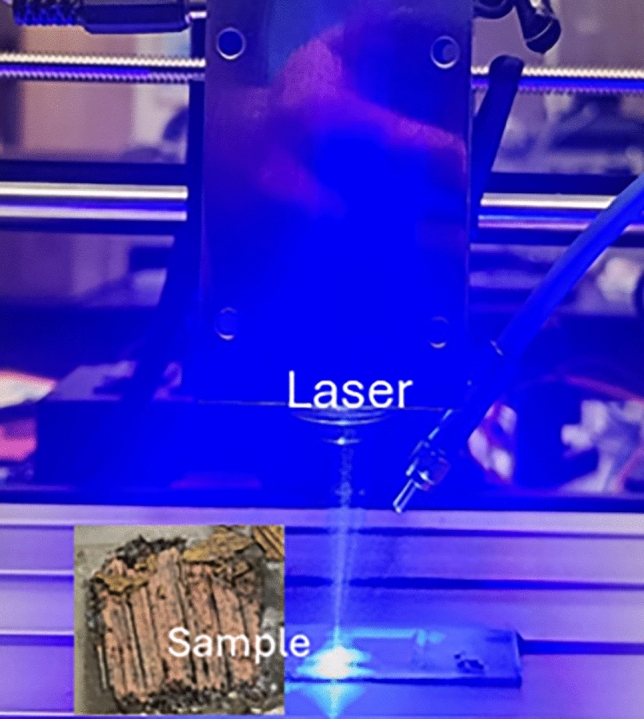

## Introduction

Nanoparticles (NPs) suspended in fluid, or nano fluids [1], have shown remarkable properties including enhanced heat transfer between liquids, mediated by the NPs. The enhanced thermal conductivity of these fluids with small-particle concentration was surprising and could not be explained by existing theories as micrometer-sized particles suspended in the liquid exhibits no such dramatic enhancement. During laser induced melting, solid powders transform into liquid. The liquid can be viewed as nanoliquids when the powder is mixed with NPs of a higher melting point so that the embedded NPs stay in solid phase but suspended in the liquid. The enhanced thermal conductivity in nano fluid can be utilized to yield better performing material properties from laser sintered materials, if the better uniformity in the composition and geometry improves the quality of laser melts during the laser melting period, which would make them more uniform in composition and geometry over larger areas. It is noted that the nano fluid can be achieved in any melting/sintering processes including the melting of lunar regolith either by a laser or concentrated sunlight on the Moon [2]. The enhanced thermal conductivity due to NPs in the powder may improve the quality of the solidified, laser melts.

Laser additive manufacturing (LAM), or 3D printing has emerged as a potent platform to acceleration materials innovation [3]. Understanding of laser melting of powers is fundamental to Additive Manufacturing (AM) that is a great way to create 3D structures at the moon if the regolith can be used as feedstock. It is noted that NPs may enhance the AM processes for 3D structures on the moon. Recently, NPs were reported to be responsible to 68% increase of the depth of melted zone (MZ) through the enhanced melting and solidification [4]. Laser printed nanocomposite delivers improved performances where the precursory matrix embedded with NPs were prepared in graphite crucible [5]. These approaches are not Powder Bed Fusion (L-PBF) method as they avoid the loss of NPs either by electrodeposition of NPs [4] or by breaking NP containing Aluminum matrix ultrasonically [5] into micrometer-sized powders, which may make the process difficult for 3D printing regolith on the moon. However, the conventional L-PBF based AM typically exposes precursory powders to air. Upon absorption of the laser energy, some are melted, and some NPs are kinetically activated so they move in random directions, and some leave off the mixture. The others may collide with the bottom surface or powders below, which induces the directional change of NP’s velocity. After several of these collisions, NP’s direction of motion will be directed upward, resulting in the additional loss of NPs from the powder bed. So those NPs leaving the powder bed may contribute nothing to the reactions happening after the absorption of energy from the energy sources. It is noted that the same argument may be applicable to almost all kinds of sintering processes that involve melting powders mixed with NPs when they are heated from the top like 3D printing. The loss of NPs makes the assessment of the impacts of NPs on the sintering/melting process difficult, if not impossible, during the period of melting and sintering of powder materials, which makes the study on NPs contributions on sintering challenging. Melting powders with NPs has not been successful in our prior experiments because of the loss of NPs during the initial period of sintering when the agitated NPs leave the substrate. This voided the merits of using NPs. We report a novel method to overcome this problem of the loss of NPs: the mixtures of powders with NPs between were placed between two slides of high melting point quartz (fused silica) glass so that the loss of NPs during laser sintering process can be reduced.

## Experiment

**Fig. 1:**
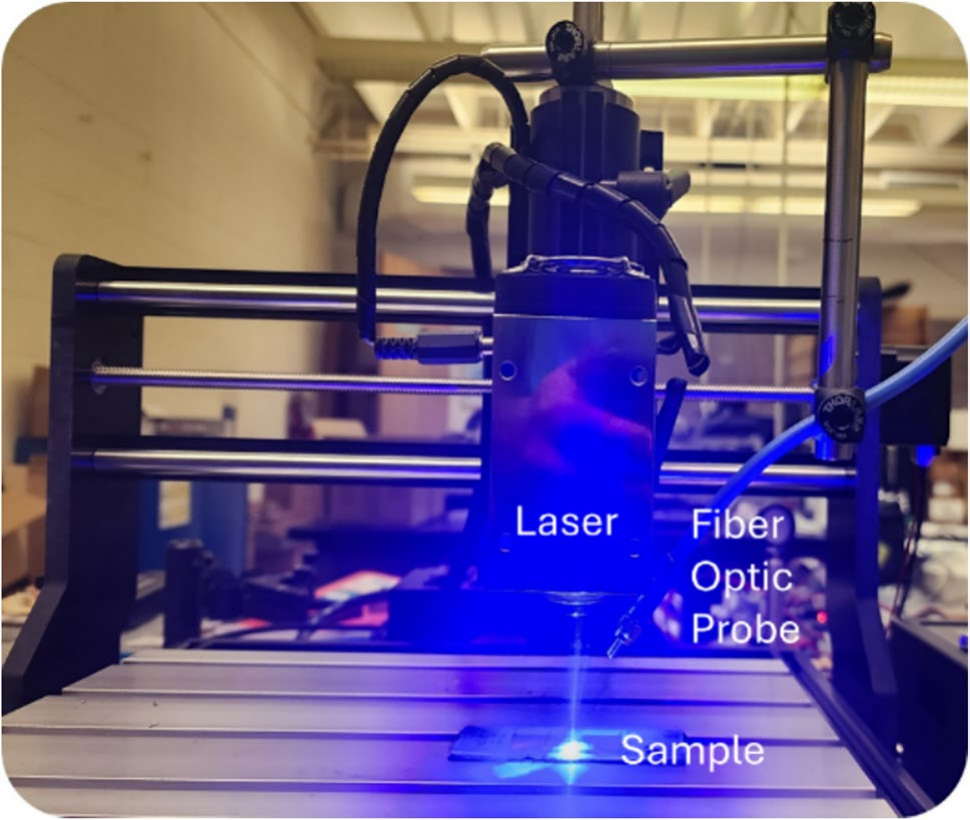
A picture of laser head mounted on a CNC machine. Laser head, sample, and fiber optic cable near the laser head are shown

**Fig. 2:**
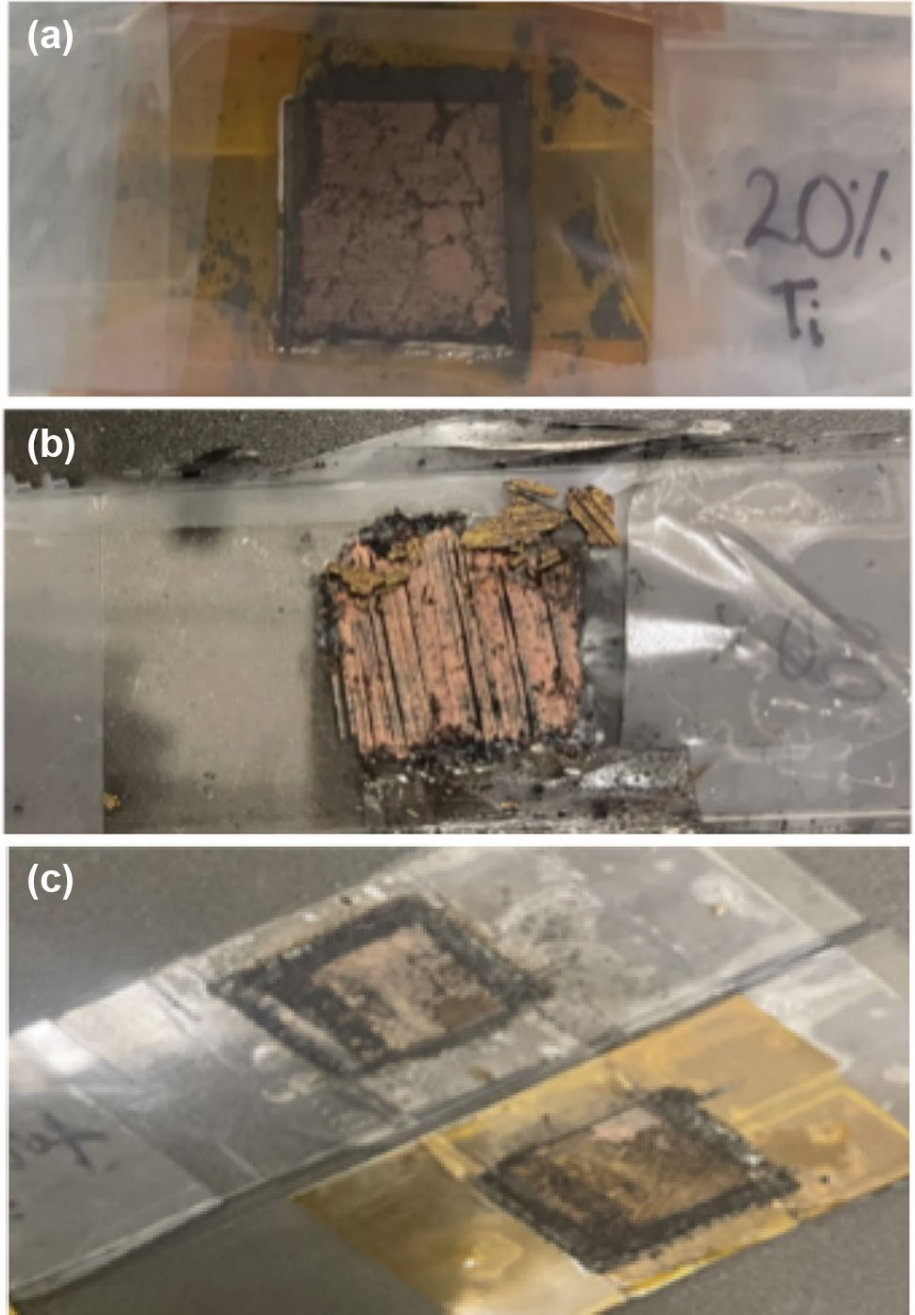
Pictures of laser sintered SmCo5 with weight percentage of Ti NPs: **a** 20%, **b** 30%, and **c** 40%. The slides are 1 inch wide and 3 inches long so that the sintered size is about 16 mm × 16 mm

Titanium NPs of average particle size (APS) of 30 nm–50 nm (American Elements, PN: TI-M-02-NP, 99 + %) were mixed with 99 + % SmCo_5_ powder (American Elements, SM-Co-021 M-P.4T7UM) of APS 4 to 7 mm. The mixtures were placed between two quartz glass slides. Some laser melts from the powders are shown in Fig. 2. Laser melting is carried out using a 10-W, continuous wave (CW) blue laser of 446 nm. This diode laser is mounted to a commercial Computer Numerical Control (CNC) machine so that laser melting or sintering is carried out automatically via computer control. A computer code was written and used to control CNC machine via a USB cable so that the sintered sample size and the speed of laser spot were controlled through the control of in x-, y-, and z-positions of the laser head. Here x- and y-directions are for the head to cover the lateral dimensions of the area and the control in z-direction is to control the laser spot size as it is the distance between the lens in the laser head and the sample. The spot size may be diffraction limited for the given laser diode, possibly between 40 and 60 mm in diameter for commercial blue colored diode lasers, according to Opt Lasers, https://optlasers.com/blue-laser. The scan speed can be controlled, typically about 0.1 cm/sec. Figure 1 shows the photo of laser head mounted to a CNC machine. A line of melted strip is realized along the x-direction when the laser head moves linearly along the direction. Assuming the powder has the same melting point of bulk SmCo_5_, the temperature of laser spot may exceed the bulk melting point of 1325 °C, according to Safety Data Sheet from ThermoFisher. Our quartz slides did not crack even after the extended period of scanning of laser spot, which took less than 10 min to cover the 1.6 cm × 1.6 cm area, possibly because the softening point (glass transition temperature) of fused silica glass is 1655 °C, which is a little lower than the melting point of “crystalline” quartz 1670 °C. After the first line of melting, the laser spot was moved along the y-direction at a small increment, perpendicular to the first line. The overlap between these two lines was controlled to yield different sintering results. The hardness of the sintered powder was measured using micro hardness tester.

## Results and discussion

Pictures of some laser melts are shown in Fig. 2. These samples were produced from the SmCo_5_ powder mixed with titanium nanoparticles with Ti NPs weight percents of 5, 10, 20, 30, and 40%. The picture of some laser melted samples with Ti NP compositions from 20% to 40% are shown in Fig. 2. To produce these melts, CNC took less than 60 min per sample. The pictures show that the color and appeared grain structures are similar regardless of the concentration of Ti NPs. The results demonstrate that the power of 10-W cw laser is strong enough to produce melts, but the melts have some irregularities such as dark lines in Fig. 2b, possibly because uneven melting and uneven thickness of powder over the area. Longer exposures with a smaller increment between two successive scan lines may be necessary.

**Fig. 3:**
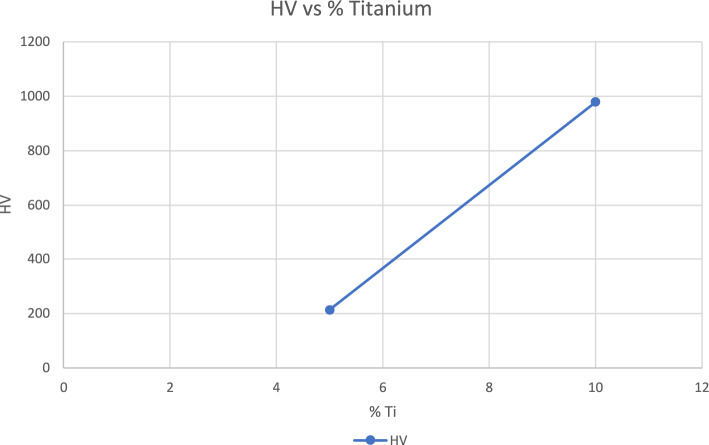
Graph showing the hardness of laser sintered SmCo_5_ with weight percentage of Ti NPs

The results of hardness testing of the laser melted SmCo_5_ powder mixed with the NP weight % of 5 and 10% are shown in Fig. 3. The higher hardness is measured with the bigger NP composition. More data will be collected with more uniform melting with compositions between 5% and 10% to confirm the trend that Vicker’s hardness in HV may increase with the weight percent of Ti NPs. It is noted that Vicker’s hardness of 5% NP is smaller than that of SmCo_5_ in bulk form, which is 500 to 600 HV [6]. Table 1 [7] shows the hardnesses of some elements. The smaller value of hardness was measured possibly because uneven melting, as shown in grains in Fig. 2b. More uniform melting may explain the hardness value measured from 10% NPs, which is larger than that of bulk SmCo_5_. The bigger hardness measurement is an exciting result because an addition of Ti NPs may cause the mixture to be harder than that of the bulk SmCo_5_. In addition, the hardness of laser melts with 10% Ti NPs is comparable to those of Titanium oxides. Hardness testing with a bigger percentage of Ti NPs have been difficult as they are too brittle to measure so far. Further study on the hardness measurements with a bigger concentration of NPs will be carried out with the focus on the uniformity in geometry including the depth of melting as well as compositional uniformity of laser melting and other alloying effects during the laser melting process.

**Table 1:**
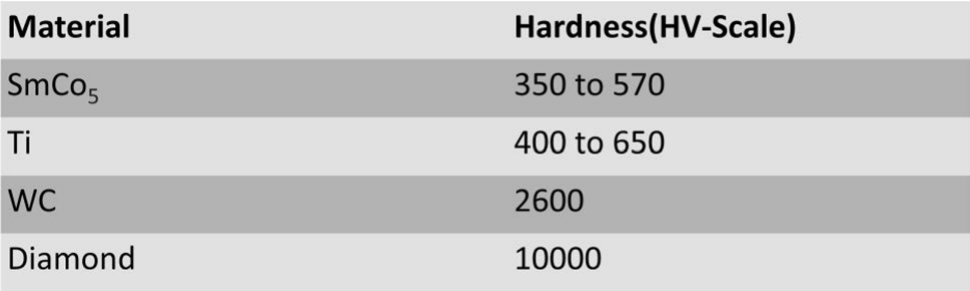
Hardness of bulk SmCo_5_ and other materials

## Conclusion

We have successfully produced laser melts SmCo_5_ powders mixed with titanium NPs without losing them so that the weight percentage of NPs can be assumed nominal fractions. Our data show that nanoparticles improve the hardness of laser melts when the fraction of Ti NPs is 10%, bigger than those of SmCo_5_ and Titanium.

## Data Availability

Available upon request. All results are obtained without any software and found by manual computations.
